# Prevalence of alcohol use disorders among under- and post-graduate healthcare students in Italy

**DOI:** 10.1371/journal.pone.0175719

**Published:** 2017-04-24

**Authors:** Monica Lamberti, Francesco Napolitano, Paola Napolitano, Antonio Arnese, Vincenzo Crispino, Gianclaudio Panariello, Gabriella Di Giuseppe

**Affiliations:** 1 Department of Experimental Medicine, Section of Occupational Medicine, School of Medicine, Second University of Naples, Naples, Italy; 2 Department of Experimental Medicine, Section of Hygiene, School of Medicine, Second University of Naples, Naples, Italy; National Institute of Health, ITALY

## Abstract

A cross-sectional study was carried out on 641 medical students, 359 students attending a degree course in the healthcare professions, and 500 resident physicians, all undergoing health surveillance at the ambulatory of the Division of Occupational Medicine, Second University of Naples, Italy. 76.1% of the participants drank alcohol, with 85.5% of medical students, 77.4% of resident physicians, and 63% of healthcare-professions students reporting regular alcohol use. In the whole sample, the mean Audit-C score was 1.6 for men and 1.1 for women; only 5.5% of men and 7.1% of women had a hazardous alcohol consumption with an Audit-C score of respectively ≥4 and ≥3. Multivariate regression modeling revealed that regular alcohol use was more likely in individuals who were men, were younger, had a lower body-mass index, were active smokers, were habitual coffee drinkers, and who were resident physicians or medical students rather than healthcare-professions students. This finding identifies a need to assess alcohol use in medical-profession workers in order to identify risky behavior early on and to carry out rapidly effective preventive and curative interventions.

## Introduction

Use of substances like alcohol has become a major rising public health and socio-economic problem worldwide. Alcohol dependence is, in fact, a major risk factor for mortality and disability [1–4], and alcohol consumption is the third leading preventable cause of death [5]. It is estimated that 9% of the global teenager population has a dependence on alcohol [6]. Excessive alcohol consumption has many physiological, social, and mental consequences, such as impaired vision and motor coordination, elevated blood pressure and heart rate, risk of stroke and heart failure, introversion, and antisocial behavior [7].

Several investigations conducted in United States and United Kingdom suggests that medical students have higher rates of drinking [8–10]. This problem may be linked to the type of activities undertaken by medical students, associated with the level of academic pressure, workload, and burnout [11–14]. Indeed, substance abuse, including over consumption of alcohol, is regarded a method of stress reduction among national and international medical students [15,16], and medical students have been reported to consume alcohol at levels exceeding the recommended guidelines [17,18].

The understanding and screening of alcohol abuse in the population and workers is very important for the prevention of risky behavior and to reduce the psychological and physical negative effects due to the hazardous assumption. Therefore, the excessive alcohol use has been extensively evaluated worldwide through the Alcohol Use Disorders Identification Test for Consumption (AUDIT-C) scale, a validated short modified version of the 10-item AUDIT instrument [19]. Indeed, previous investigations using the AUDIT-C scale have screened the alcohol consumption in different fields and in particular among the general population [20,21], university students [22,23] and physicians [24,25].

The present study was therefore carried out to assess the prevalence of alcohol drinking in a large sample of students and resident physicians attending the School of Medicine of the Second University of Naples, and to evaluate the factors associated with this outcome of interest.

## Materials and methods

A cross-sectional study was carried out on a sample of 641 medical students, 359 students attending a degree course in the healthcare professions at the School of Medicine, and 500 resident physicians undergoing a health-surveillance visit at ambulatory of the Division of Occupational Medicine, Second University of Naples. Students in their third and sixth year of medical school, healthcare-professions students in their first and third year, and students of specializing medical schools in their first and third year were actively required to undergo Alcohol Use Disorders screening. The students of the health-professions schools were nursing students, pediatric nursing students, student radiographers and midwifery students; the students of specializing schools were post-medical school students.

The sample size was calculated based on an expected rate of the population with regular alcohol use of 80% in according with the literature, a confidence level of 95% and an accepted precision of 5%. The required sample size was estimated to be of 250 students. In order to select a representative sample, assuming a 40% for non-response rate, the final sample size was calculated to be approximately of 350 participants. The total number of students and resident physicians who volunteered agreed to participate in the study was 1500.

Before starting the survey, written informed consent was obtained from each participant. Data collection was performed between March and December 2015 by four trained physicians using a survey form that allowed to collect data through consultation of the medical records of all participants undergoing a medical examinations in 2014. The items of the form have been chosen based on the demographic and clinical information in the medical records, according to previous studies in the literature [22,26,27] and assuming potential predictors of the alcohol use. In particular the following information was collected: (a) socio-demographic characteristics (age, gender, academic year, marital status, body mass index); (b) lifestyle behavior (smoking status, coffee/alcohol consumption, and physical activity); and (c) medical history (number and type of diseases, medication use).

Alcohol-related disorders were investigated using the Audit-C scale, a validated and effective questionnaire for predicting hazardous alcohol drinking [21,28–31], as prescribed by Italian legislative decree n. 81; it was administered to the participants after a medical examination at the ambulatory. The Audit-C questionnaire employed was a shortened version incorporating only the first three questions: 1) How often do you consume alcoholic drinks?; 2) On days when you drink, how many alcoholic drinks do you consume on average?; 3) How often do you drink six or more glasses of alcohol on a single occasion? The score for each answer went from 0 to 4, and the final score of the Audit-C questionnaire ranged from 0 to 12. The test reveals an above-average risk of developing an alcohol-related disorder (consumer at risk, harmful consumption, or alcohol dependence) upon a total score of 4 points or more for a male, and three points or more for a female.

A pilot study was conducted on a random sample of 25 medical records in order to evaluate the validity, reliability, and completeness of the instrument for data collection. Before starting the study, ethical approval was obtained from the Ethics Committee of the Second University of Naples, which reviewed the proposal, the survey form, and the consent form of the study.

### Statistical analysis

The statistical analysis of data was divided into two stages: descriptive analysis and inferential analysis. For the former, all information was synthesized in tabular form; the latter provided the use of techniques of bivariate analysis, in particular Student’s t-test for continuous variables and chi-square test for categorical variables. Moreover, stepwise multivariate logistic regression analysis was performed, with p-values of 0.2 and 0.4 for the inclusion and elimination of the variables in the model, to explore independent characteristics associated with the outcome of interest (profile of regular alcohol drinkers). The results of logistic regression models were reported as odds ratio (OR), relative confidence intervals (CI) at 95%, and p-value. In the model, the following independent variables were included: gender (male = 0; female = 1), age, (continuous), participant’s group (students of a degree courses in the healthcare professions = 0; medical students and resident physicians = 1), body mass index (underweight = 1; normal weight = 2; overweight = 3; obesity = 4); blood pressure (continuous); smoking status (non-smoker = 0; regular smoker = 1); habitual coffee consumption (no = 0; yes = 1); chronic diseases (no = 0; yes = 1); medication use (no = 0; yes = 1). All inferential tests were performed by the execution of bilateral hypothesis test with a level of statistical significance (p-value) set at ≤0.05. The statistical software package Stata version 10.1 was used to carried out the analysis [32].

## Results

Socio-demographic characteristics and lifestyles are given in Table 1. All individuals provided consent for participation and completed the Audit-C questionnaire, with a response rate of 100%. More than half of participants were female, the mean age was around 26 years, and almost all were unmarried. Two-thirds of the sample had a normal weight and just over 4% were obese, according to body mass index. Regarding the health of the participants, just under 16% had at least one chronic disease, and slightly more than 13% were taking medication.

**Table 1:** Socio-demographic characteristics and lifestyle habits of the participants.

	Total (n = 1500)		Medical students (n = 641)		Healthcare-professions students (n = 359)		Resident physicians (n = 500)	
	N	%	N	%	N	%	N	%
Gender								
Male	613	40.9	287	44.7	118	32.9	208	41.6
Female	887	59.1	354	55.2	241	67.1	292	58.4
Age (years)	26.2±5.5(19–54)^a^		23.9±3.5(19–49)^a^		22.4±4.1(19–54)^a^		31.6±3.7(24–52)^a^	
Body mass index	23.1±3.5(15.8–50.8)^a^		22.8±3.3(15.8–36.6)^a^		23.4±3.8(16.1–50.8)^a^		23.2±3.4(16.3–38.1)^a^	
Underweight	70	4.7	43	6.8	6	1.7	21	4.3
Normal weight	1067	72.4	454	72.1	264	74.6	349	71.2
Overweight	275	18.6	111	17.6	66	18.6	98	20
Obesity	62	4.3	22	3.5	18	5.1	22	4.5
Regular alcohol use								
No	355	23.9	111	17.5	131	37	113	22.6
Yes	1131	76.1	522	85.5	223	63	386	77.4
Smoking status								
No	1168	78.3	533	83.4	271	77	364	72.8
Yes	323	21.7	106	16.6	81	23	136	27.2
Habitual coffee consumption								
No	155	10.4	55	8.6	61	17.1	39	7.8
Yes	1339	89.6	583	91.4	295	82.9	461	92.2
Medication use								
No	1299	86.6	553	83.1	321	89.4	445	89
Yes	201	13.4	108	16.9	38	10.6	55	11
Chronic disease								
No	1265	84.3	521	81.3	315	87.7	429	85.8
Yes	235	15.7	120	18.7	44	12.3	71	14.2

Number for each item may not add up to total number of study population due to missing value

^a^Mean±standard deviation (range)

Regarding alcohol use, just over two-thirds of the participants drank alcohol regularly, with medical students being the more likely to drink than resident physicians or healthcare-professions students. The mean Audit-C score (Table 2) for the whole sample was 1.9 for men and 1.6 for women. 5.5% of men and 7.1% of women were deemed to have a hazardous alcohol consumption (Audit-C score, respectively, ≥4 and ≥3). In particular, the proportion of participants with a hazardous alcohol consumption was higher for resident physicians (7.2% of men and 9.6% of women). Regarding the other lifestyle habits, nearly 90% habitually drank coffee, one in five were active smokers, and less than 60% performed physical activity at least occasionally.

**Table 2:** Audit-C score for the study population.

	Audit-C score											
	0		1		2		3		4		5	
Medical students	**N**	**%**	**N**	**%**	**N**	**%**	**N**	**%**	**N**	**%**	**N**	**%**
Men	43	15	97	33.8	110	38.3	25	8.7	12	4.2	0	0
Women	72	20.4	164	46.3	91	25.7	21	5.9	6	1.7	0	0
Healthcare-professions students												
Men	26	22	42	35.6	34	28.8	9	7.6	6	5.2	1	0.8
Women	106	44	81	33.6	46	19.1	7	2.9	0	0	1	0.4
Resident physicians												
Men	28	13.5	50	24.1	88	42.3	27	13	12	5.8	3	1.4
Women	89	30.4	88	30.2	87	29.8	23	7.9	4	1.4	1	0.3
All participants												
Men	97	15.9	189	30.8	232	37.9	61	9.9	30	4.9	4	0.6
Women	267	30.1	333	37.5	224	25.3	51	5.8	10	1.1	2	0.2

Table 3 gives the results of univariate and multivariate regression analyses built to investigate the variables associated with the outcome of interest. At univariate analysis, four variables (gender, participant’s occupation, smoking status, and habitual coffee consumption) were associated with the profile of regular alcohol drinking. The results of multivariate regression revealed that regular alcohol use was more likely in men and in younger individuals, in those who had a lower body-mass index, in those who were active smokers, in those who habitually drank coffee, and in resident physicians and medical students than in healthcare-professions students.

**Table 3:** Univariate and multivariate analyses exploring the characteristics associated with the profile of regular alcohol drinkers.

	Univariate	Multivariate	
	p	OR (95% CI)	p
Gender	<0.001	0.37 (0.27–0.5)	<0.001
Age	0.664	0.97 (0.94–0.99)	0.023
Participant group ^a^	<0.001	2.38 (1.74–3.25)	<0.001
Body mass index	0.363	0.94 (0.91–0.97)	0.001
Smoking status	<0.001	2.34 (1.63–3.36)	<0.001
Habitual coffee consumption	<0.001	2.54 (1.74–3.69)	<0.001
Medications use	0.555	1.34 (0.91–1.99)	0.142
Chronic diseases	0.666	-	-

^a^Healthcare professions students vs. medical students and resident physicians.

## Discussion

To our knowledge this is the first study carried out in Italy who evaluated the alcohol use and the alcohol hazardous consumption using the Audit-C scale both in medical students and resident physicians and that assessed which factors could be associated with regular alcohol use of participants.

Current Italian legislation obliges employers to prevent workers, such as healthcare personnel, who are occupationally at risk or who perform jobs that may be hazardous for the safety or health of third parties, from consuming alcohol. Medical students and assistants in training are assimilated, according to Italian law, with health professionals (Dgls. no. 81/08). This study was therefore carried out to assess alcohol use in a large sample of students and physicians in Italy, with the aid of the Alcohol Use Disorders Identification Test for Consumption (Audit-C) scale, which is an effective screening test for the full spectrum of alcohol misuse (including alcohol-use disorders and risky drinking), as well as for alcohol-use disorders alone.

We found that three-quarters of respondents reported regular alcohol use. This result is comparable with the findings of studies conducted in Germany, in which 82.5% of physicians and 62.8% of medical students reported usual alcohol use [33], and in the USA, in which 78% of medical students reported alcohol consumption in the previous month [34]. Conversely, the proportion of participants drinking alcohol in our sample was lower compared with studies conducted among university students in Sweden (91%) and New Zealand (93%) [35,36].

In our study, the mean Audit-C score was low (1.7), and very few participants self-reported risky behavior related to alcohol use (only 5.5% of men and 7.1% of women). This is lower than those reported in other studies conducted with the Audit-C scale, albeit with different cut-off values for hazardous alcohol consumption and different methodologies: indeed, in a study conducted among university students in Ireland, 17% of men and 5% of women had an Audit-C score of 10 or higher [22], and in Finland a survey found that 24% of women and 49% of men among medical students had a risk behavior related to alcohol (Audit-C values equal or greater than 5 and 6, respectively) [26]. In another study conducted with a 10-item Audit scale on Australian doctors, 15% of participants reported hazardous alcohol use (8% of women and 17% of men) [24].

Upon univariate and multivariate logistic regression analyses, several factors were found associated with the outcome of interest: these were age, gender, smoking status, and habitual coffee consumption. Indeed, an interesting finding was that men and younger participants were more likely to be regular alcohol drinkers. This is in accordance with previous studies finding that male medical students drank more often, more intensely, and in larger amounts than female medical students [27].

The study has several limitations that could affect the generalizability of the results. The first is that the causal direction between the outcome and the investigated associated factors in the cross-sectional study cannot be determined. The second is that risky alcohol use may be underestimated by the Audit-C questionnaire administered to students and physicians during an occupational medical examination. Finally, the sample of students and resident physicians may not be representative of the target populations due to the fact that participants were recruited from a single center. However, the participants not differ significantly from the students’ and physicians’ population of Campania Region and of Italy with respect to the main socio-demographic characteristics. Moreover, the sample was very large, the response rate was 100%, the data were collected carefully and results were valid.

In conclusion, the results of this study refer to the need of assessing alcohol-use behavior in students of the healthcare professions and in resident physicians in order to identify risky behavior early and to carry out rapidly effective preventive and curative interventions.
